# Factors Predicting Acute Brain Injury in Cases of Carbon Monoxide Poisoning: A Prospective Registry-Based Study

**DOI:** 10.3390/toxics9060120

**Published:** 2021-05-27

**Authors:** Hoon Lim, Young Hwan Lee, Sangun Nah, Sungwoo Choi, Young Soon Cho, Gi Woon Kim, Ji Eun Moon, Sangsoo Han

**Affiliations:** 1 Department of Emergency Medicine, Soonchunhyang University Bucheon Hospital, Bucheon 14584, Korea; emhoney6714@gmail.com (H.L.); emer0716@naver.com (Y.H.L.); potter325@naver.com (S.N.); csw3613@naver.com (S.C.); emer0717@gmail.com (Y.S.C.); emer0325@naver.com (G.W.K.); 2 Department of Biostatistics, Clinical Trial Center, Soonchunhyang University Bucheon Hospital, Bucheon 14584, Korea; moon6188@schmc.ac.kr

**Keywords:** acute brain injury, carbon monoxide poisoning, magnetic resonance imaging

## Abstract

Carbon monoxide (CO) is one of the most common poisoning substances worldwide. Since acute brain injury (ABI) is an important determinant of the neurological outcome in CO poisoning, screening for patients at a high risk of developing ABI is essential for the proper treatment. This study identified predictors of ABI in patients with CO poisoning. This prospective registry-based study was conducted in patients who visited a tertiary care hospital for CO poisoning from August 2016 to June 2020. ABI was defined as the presence of acute hypoxic lesions on diffusion-weighted magnetic resonance imaging. Multiple logistic regression analysis was performed to identify the predictors of ABI. Of 231 patients, 64 (27.7%) showed ABI. Multiple logistic regression analysis showed that a Glasgow Coma Scale (GCS) score <9 at presentation (odds ratio [OR] 3.28, 95% confidence interval (CI) 1.08–10.01), creatinine level >1.2 mg/dL (OR 3.04, 95% CI 1.16–8.01), and C-reactive protein (CRP) level >9.2 mg/L (OR 4.38, 95% CI 1.41–13.65) predicted ABI in cases of acute CO poisoning. In CO poisoning, the GCS score at presentation, and serum creatinine and CRP levels, were useful predictors of ABI, and may help clinicians identify high-risk patients for whom treatment should be prioritized.

## 1. Introduction

Carbon monoxide (CO) poisoning is common, being responsible for more than 50,000 emergency department (ED) visits per year in the United States (16 cases per 100,000 population) [1]. CO is a colorless, tasteless, and odorless gas generally produced by incomplete combustion of carbon compounds. Common sources include fire, engine exhaust, and furnace defects [2]. It is also used by some individuals when attempting suicide; about 15,000 cases of deliberate CO poisoning are reported annually in the United States, of which two-thirds result in death [3].

CO competes with oxygen for the binding site on the heme portion of hemoglobin (Hb) and CO bound with Hb forms carboxyhemoglobin (COHb). However, CO has 250 times greater affinity than oxygen; thus, even small amounts of CO can lead to severe tissue hypoxia [4]. CO also binds to myoglobin and mitochondrial cytochrome oxidase, and may cause injury through inflammation-induced brain lipid peroxidation [5]. Organs with high dependence on oxygen and high metabolic rates, such as the brain and heart, are more susceptible to CO [6,7]. Acute brain injury (ABI) caused by CO poisoning is associated with neurological sequelae. ABI can be confirmed by diffusion-weighted magnetic resonance imaging (DW-MRI) [8].

However, due to cost and time constraints, it is difficult to perform DW-MRI in all CO poisoning patients. DW-MRI can also be risky, especially in people with unstable vital signs or on ventilator care. Therefore, it is critical to identify risk factors for ABI before DW-MRI is performed in CO poisoning patients. In this study, we identified predictors of ABI in CO poisoning patients.

## 2. Materials and Methods

### 2.1. Study Design

This prospective registry-based study was performed at the ED of an urban tertiary care hospital (Bucheon, Korea) that has more than 65,000 annual visits. All people visiting the ED for CO poisoning have been registered on the CO registry since 2016. CO poisoning was considered in the following cases: COHb level ≥5% in non-smokers (≥10% in smokers) at presentation or if there is obvious evidence of CO poisoning in history [9].

### 2.2. Study Population and Measures

This study included all acute CO poisoning patients admitted to the ED between August 2016 and June 2020. The exclusion criteria were as follows: <18 years of age, discharged against medical advice, and DW-MRI not performed. 

The following clinical characteristics of the patients were obtained from the CO registry: sex, age, body mass index (BMI), vital signs, comorbidities, smoking status, exposure type (accidental or intentional), Glasgow Coma Scale (GCS) score, duration of CO exposure, symptoms (headache, loss of consciousness, dyspnea, and chest pain), laboratory findings (COHb, white blood cell (WBC) count, blood urea nitrogen (BUN) level, creatinine level, creatine kinase level, pH, C-reactive protein (CRP) level, lactate and troponin I levels), and length of stay (LOS) at the hospital. Cutoff values for GCS score, WBC count, creatinine and creatine kinase level were 9, 10,000/μL, 1.2 mg/dL and 1000 U/L, respectively [10,11,12].

DW-MRI was performed to examine ABI, which was defined as a newly developed brain lesion on DW-MRI [8,13]. All images were read by a radiologist blinded to the patient’s information. DW-MRI was performed with a 3-T MRI unit (Signa HDxt 3.0; GE Healthcare, Chicago, IL, USA) using a standard head coil. The DW-MRI parameters were as follows: repetition time, 9000 ms; echo time, 77.5 ms; matrix, 192 × 192; field of view, 200 mm; 2 b values of 0 and 1000 s/mm^2^; slice thickness, 5 mm; and interslice gap, 1 mm. Diffusion-weighted images and the automatically generated apparent diffusion coefficient map were studied (b = 1000 s/mm^2^).

### 2.3. Treatment Protocol

All patients who presented with CO poisoning were immediately treated with normobaric oxygen (NBO) therapy. If the case met the indication, hyperbaric oxygen (HBO) therapy was then administered within 1 h of the visit. Otherwise, NBO therapy was performed until the symptoms, such as headache, dyspnea, chest pain and altered mental status disappeared and the COHb level drops to <5%. The indications for HBO therapy in our institution are as follows: COHb level ≥25%; neurologic deficits, such as altered mental state, impaired cognitive function, loss of consciousness or seizure, and/or suspicious myocardial ischemia, such as chest pain or elevated cardiac enzymes. In the HBO therapy protocol, three sessions were conducted within 24 h: the first session was conducted for 150 min at 3 atmospheres absolute (ATA), and the second and third sessions were conducted for 120 min at 2 ATA [14]. DW-MRI was performed immediately after one round of the protocol was finished.

### 2.4. Statistical Analysis

IBM SPSS Statistics for Windows software (version 26.0; IBM Corp., Armonk, NY, USA) was used for all statistical analyses in this study. Continuous variables are expressed as mean ± standard deviation (SD) for normally distributed data (as indicated by the Shapiro–Wilk test), and as median with interquartile range (IQR) for non-normally distributed data. Categorical variables are expressed as absolute numbers with percentages. To compare two groups, Student’s t-test and the Mann–Whitney U test were used for normally and non-normally distributed continuous variables, respectively. For categorical variables, the χ2 test or Fisher’s exact test was used. Cutoff values for BUN and CRP were determined using Youden’s index. Univariable logistic regression was used to screen out variables, and we selected those with overall *p*-values < 0.15 as candidate variables for the multivariable analysis. Multiple logistic regression analysis was used to find out predictors of ABI, and the results are expressed as the odds ratio (OR) with the 95% confidence interval (CI). In addition, the area under the receiver operating characteristic curve (AUC) was calculated to determine model performance. *p* < 0.05 was considered statistically significant, and all *p*-values were two-sided.

## 3. Results

**Figure 1:**
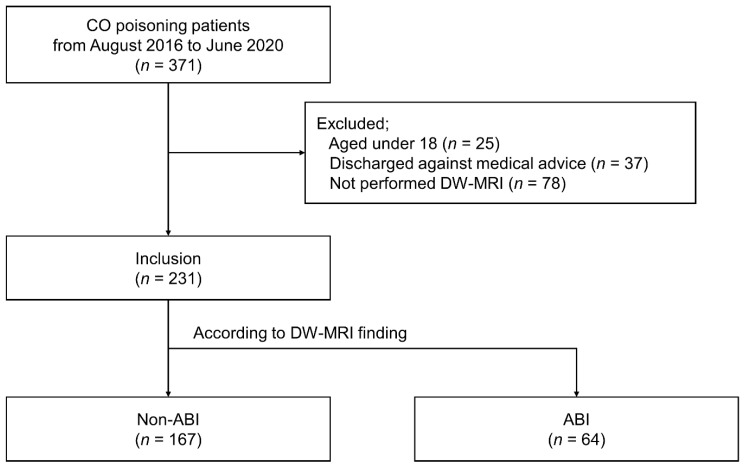
Flow diagram of the study procedures. ABI, acute brain injury; CO, carbon monoxide; DW-MRI, diffusion-weighted magnetic resonance imaging.

A total of 371 CO poisoning patients were identified during the study period. We excluded 140 patients according to the following criteria: < 18 years of age (25 patients), discharged against medical advice (37 patients), and DW-MRI not performed (78 patients). Thus, a total of 231 patients were enrolled and categorized into non-ABI (167 patients, 72.3%) and ABI groups (64 patients, 27.7%) (Figure 1).

### 3.1. Baseline Characteristics

Table 1 summarizes the baseline clinical characteristics. The median age of the patients was 43 years, and 88 (38.1%) were female. In total, 23 patients (10%) had hypertension, 7 (3%) had diabetes, and 86 (38.1%) were current smokers. The median GCS score on presentation at the ED was 15. CO exposure was intentional in 162 people (70.1%). The median exposure time was 180 min, and 224 patients (97%) received HBO therapy.

### 3.2. Comparison of Clinical Characteristics and Laboratory Findings Based on the Presence of ABI

The non-ABI and ABI groups differed significantly in terms of median age (40 vs. 48 years; *p* < 0.001), CO exposure time (120 vs. 240 min; *p* = 0.045), GCS score at presentation (15 vs. 13; *p* < 0.001), CO exposure type (intentional exposure, 110 vs. 52 patients; *p* = 0.034), WBC count (11.5 vs. 14.82 10^3^/μL; *p* < 0.001), BUN level (13.79 vs. 21.01 mg/dL; *p* < 0.001), creatinine level (0.98 vs. 1.36 mg/dL; *p* = 0.013), creatine kinase level (617.96 vs. 5390.73 U/L; *p* = 0.033), CRP level (3.8 vs. 31.8 mg/L, *p* < 0.001), and median LOS at the hospital (3 vs. 7 days; *p* < 0.001) (Table 2).

### 3.3. Predictors of ABI in Cases of Acute CO Poisoning

**Figure 2:**
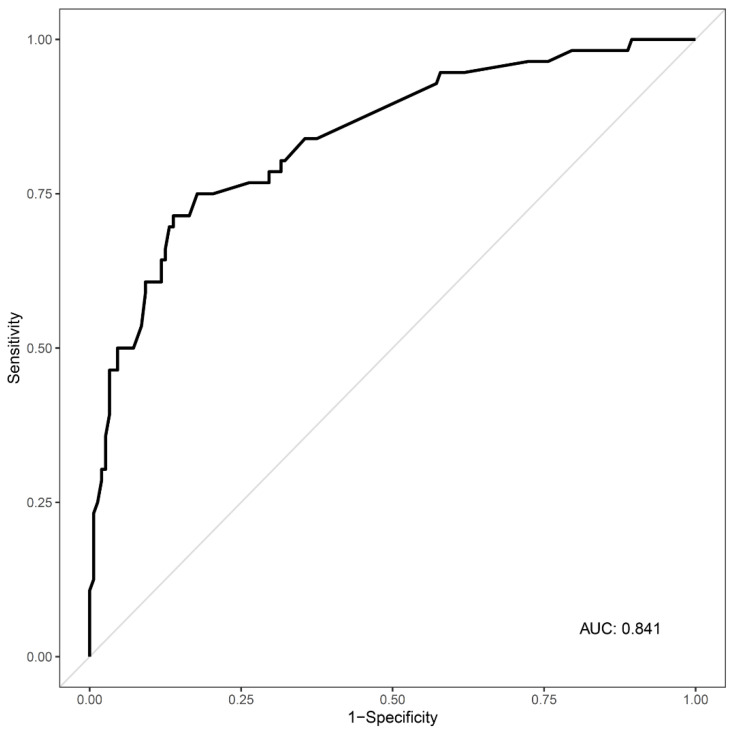
Receiver operating characteristic curve for the multiple regression model. AUC = 0.841 (95% confidence interval, 0.778–0.904). AUC, area under the curve.

The results of univariable logistic regression analysis are shown in Supplemental Table S1. The GCS score at presentation, creatinine level, and CRP level were analyzed by multiple logistic regression to identify predictors of ABI in cases of acute CO poisoning. A GCS score <9 (OR 3.28, 95% CI 1.08–10.01, *p* = 0.037), creatinine level >1.2 mg/dL (OR 3.04, 95% CI 1.16–8.01, *p* = 0.024) and CRP level >9.2 mg/L (OR 4.38, 95% CI 1.41–13.65, *p* = 0.011) were significant predictors of ABI. Since all of the variance inflation factors (VIF) for the factors were under 10, there was no multicollinearity among the independent variables (Table 3). The AUC for the multiple logistic regression model was 0.841 (95% CI 0.778–0.904) (Figure 2).

## 4. Discussion

In this study, DW-MRI indicated ABI in 27.7% (64/231) of patients with acute CO poisoning. A GCS score <9 at presentation (OR 3.28), serum creatinine level >1.2 mg/dL (OR 3.04), and CRP level >9.2 mg/L (OR 4.38) were strong predictors of ABI.

Higher CO exposure levels and longer-duration exposure are associated with more severe symptoms and a greater likelihood of developing ABI [5]. Therefore, ascertaining the exact exposure duration, and the CO level at the time of exposure, can greatly facilitate patient evaluation. Unfortunately, most cases of CO poisoning in Korea are due to suicide attempts; such patients are not only uncooperative in terms of history taking, they also frequently do not know the exposure duration [15,16]. In this study, 70.1% (162/231) of patients were admitted to the hospital due to a suicide attempt, and only 40.3% (93/231) provided an exposure time. In addition, it is difficult to accurately estimate the CO level during exposure because patients generally do not visit the hospital immediately after being exposed, and usually receive oxygen in an emergency medical service vehicle en route. Therefore, it is more desirable to evaluate CO poisoning patients objectively based on findings such as GCS scores, vital signs, symptoms, and laboratory results.

Previous studies have suggested that the GCS score at presentation is closely related to the severity and prognosis of CO poisoning [17,18]. O’Donnell et al. reported that 63.2% of patients who lost consciousness after CO poisoning had brain MRI lesions [19]. A recent study showed that a GCS score <9 was a significant independent predictor of acute brain lesions on MRI [20]. Similarly, in this study, a GCS score <9 was a strong predictor of ABI.

CO can cause inflammation through mechanisms such as increasing levels of cytosolic heme and heme oxygenase-1 protein, and greater exposure can increase inflammation severity [5]. Grieb et al. reported that the CRP and leucocyte count were useful tools for evaluating the severity of CO-related illness [17]. Additionally, high CRP levels were reported to be a risk factor for neurological sequelae [21]. In our study, a CRP level >9.2 mg/L was also found to be a significant predictor of ABI, whereas WBC count showed no association. CO poisoning can damage the kidneys; thus, the creatinine concentration, an indicator of kidney damage, can reflect the severity of CO poisoning [22]. CO causes hypoxic kidney damage by shifting the Hb dissociation curve to the left, and can also directly damage the kidney at the cellular level regardless of hypoxia [5,23]. We found that a creatinine level >1.2 mg/dL was a strong predictor for ABI.

This study had several limitations. First, it used a single-center design, so it is difficult to generalize our findings to other institutions. Second, although the treatment protocol at our institution was applied consistently, there may have been differences in prehospital treatment among cases. In South Korea, emergency medical technicians generally provide a 100% nonrebreather face mask to the patient during transfer, but we did not note the specific treatment details or the time taken to arrive at the hospital. Third, we did not consider the possible effect of the co-ingested drugs, such as antidepressants, antipsychotics, and alcohol, which the patients might have used before or during suicide attempts. It is reported that suicide attempts are frequently preceded by the use of alcohol or psychotropic agents [24,25]. Alcohol and drugs, such as antidepressants, antipsychotics, and hypnotics, can sedate the patients and contribute to the patients’ decreased mental status; therefore, the effect of CO poisoning in such patients could be overestimated. A well-designed, large-scale study is needed to address these limitations.

## 5. Conclusions

We found that the GCS score at presentation, creatinine level, and CRP level were significant predictors of ABI in CO poisoning patients. Thus, we suggest that DW-MRI can be used to confirm ABI in patients who present to the ED with a GCS score <9, creatinine level >1.2 mg/dL, and/or CRP level >9.2 mg/L.

## Figures and Tables

**Table 1:** Patient demographics.

	Total
	(*n* = 231)
Age, years	43 (32–54)
Sex, *n* (%)	
Female	88 (38.1)
Male	143 (61.9)
BMI, kg/m^2^	23.44 (21.1–25.83)
Comorbidities, *n* (%)	
Hypertension	23 (10.0)
Diabetes	7 (3.0)
Current smoker, *n* (%)	86 (38.1)
GCS score at presentation	15 (12–15)
Exposure type of CO, *n* (%)	
Accidental	69 (29.9)
Intentional	162 (70.1)
Exposure time of CO, min	180 (62.5–360)
Hyperbaric oxygen therapy, *n* (%)	224 (97.0)
LOS on hospital, days	3 (2–5)

Values are expressed as the median (interquartile range) or number (proportion). BMI, body mass index; CO, carbon monoxide; GCS, Glasgow Coma Scale; LOS, length of stay.

**Table 2:** Comparison of variables between non-ABI and ABI groups.

	Non-ABI	ABI	*p*-Value
	(*n* = 167)	(*n* = 64)	
Age, years	40 (30–50.5)	48 (39–57)	<0.001
Age group, *n* (%)			0.010
<20 years	14 (93.3)	1 (6.7)	
20–39 years	74 (80.4)	18 (19.6)	
40–59 years	61 (62.9)	36 (37.1)	
≥60 years	18 (66.7)	9 (33.3)	
Sex, *n* (%)			0.383
Female	67 (75.9)	21 (24.1)	
Male	100 (69.9)	43 (30.1)	
BMI, kg/m^2^	23.52 (21.01–26.05)	23.05 (21.21–25.39)	0.575
Comorbidities, *n* (%)			
Hypertension	18 (78.3)	5 (21.7)	0.668
Diabetes	4 (57.1)	3 (42.9)	0.400
Current smoker, *n* (%)	58 (67.4)	28 (32.6)	0.281
Vital Signs			
Systolic blood pressure, mmHg	130 (120–145.5)	127 (110–140)	0.075
Diastolic blood pressure, mmHg	80 (75–90)	80 (70–90)	0.180
Heart rate, /min	89 (77.5–102)	91.5 (83–101.25)	0.224
Respiratory rate, /min	20 (18–20)	20 (19–20)	0.574
Exposure type of CO, *n* (%)			0.034
Accidental	57 (82.6)	12 (17.4)	
Intentional	110 (67.9)	52 (32.1)	
Exposure time of CO, min *	120 (40–198.75)	240 (120–450)	0.045
GCS score at presentation	15 (12.5–15)	13 (9–15)	<0.001
GCS score < 9 (%)	9 (39.1)	14 (60.9)	<0.001
Symptoms, *n* (%)			
Headache	27 (84.4)	5 (15.6)	0.152
Loss of consciousness	33 (67.3)	16 (32.7)	0.489
Dyspnea	6 (85.7)	1 (14.3)	0.677
Chest pain	2 (50.0)	2 (50.0)	0.308
Laboratory findings			
COHb, %	12.69 ± 11.11	10.14 ± 9.19	0.093
White blood cell count, 10^3^/μL	11.5 ± 4.84	14.82 ± 6.56	<0.001
BUN, mg/dL	13.79 ± 5.43	21.01 ± 8.82	<0.001
Creatinine, mg/dL	0.98 ± 0.23	1.36 ± 1.16	0.013
Creatine Kinase, U/L	617.96 ± 2195.53	5390.73 ± 16,845.19	0.033
pH	7.41 ± 0.08	7.39 ± 0.09	0.177
C-reactive protein, mg/L	3.5 ± 8.7	31.8 ± 52.6	<0.001
Lactate, mg/dL	2.93 ± 2.17	3.55 ± 2.88	0.154
Troponin I, ng/mL	25.61 ± 271.91	11.26 ± 69.92	0.567
Hyperbaric oxygen therapy, *n* (%)	162 (72.3)	62 (27.7)	>0.999
LOS at hospital, days	3 (2–4)	7 (3–14)	<0.001

Values are expressed as the mean ± standard deviation, median [interquartile range], or number (proportion). ABI, acute brain injury; BMI, body mass index; BUN, blood urea nitrogen; CO, carbon monoxide; COHb, carboxyhemoglobin; GCS, Glasgow Coma Scale; LOS, length of stay.* Exposure time of CO: Data regarding the duration of exposure to CO were available for 93 patents (26 patients in the ABI group and 67 patients in the non-ABI group).

**Table 3:** Multiple logistic regression analysis of factors predicting acute brain injury.

	Odds Ratio	95% CI	*p*-Value	VIF
Age group				
<20 years	1			
20–39 years	2.24	0.22–23.22	0.499	8.901
40–59 years	3.90	0.38–39.87	0.251	9.231
≥60 years	1.99	0.15–25.91	0.598	4.182
SBP ≥ 140 mmHg	0.54	0.23–1.29	0.165	1.165
GCS score < 9	3.28	1.08–10.01	0.037	1.050
Intentional exposure	1.45	0.53–3.95	0.469	1.217
Headache	0.97	0.24–3.87	0.963	1.123
Laboratory findings				
WBC count > 10 × 10^3^/μL	2.46	1.00–6.06	0.051	1.066
BUN > 17.71 mg/dL	2.50	0.99–6.30	0.052	1.360
Creatinine > 1.2 mg/dL	3.04	1.16–8.01	0.024	1.089
C-reactive protein > 9.2 mg/L	4.38	1.41–13.65	0.011	1.400
Creatine kinase > 1000 U/L	1.80	0.59–5.45	0.299	1.386

BUN, blood urea nitrogen; CI, confidence interval; GCS, Glasgow Coma Scale; MRI, magnetic resonance imaging; SBP, systolic blood pressure; VIF, variance inflation factor; WBC, white blood cell.

## Data Availability

The data presented in this study are available on request from the corresponding author. The data are not publicly available due to our data contains content of personal history (sensitive patient information).

## Supplementary Materials

The following are available online at https://www.mdpi.com/article/10.3390/toxics9060120/s1.
