# Supplementary material for: Factors Predicting Acute Brain Injury in Cases of Carbon Monoxide Poisoning: A Prospective Registry-Based Study
Source: Toxics. 2021 May 27;9(6):120. doi: 10.3390/toxics9060120 (PMC8229430; doi:10.3390/toxics9060120)
Supplement: Supplementary file 1 [file toxics-09-00120-s001.zip › toxics-1177043-supplementary.pdf]

# Factors Predicting Acute Brain Injury in Cases of Carbon Monoxide Poisoning: A Prospective Registry-Based Study

**Hoon Lim, Young Hwan Lee, Sangun Nah, Sungwoo Choi, Young Soon Cho, Gi Woon Kim, Ji Eun Moon, Sangsoo Han**

**Table S1.** Univariable logistic regression analysis for the factors predicting acute brain injury.

| | **Odds Ratio** | **95% CI** | **P-Value** |
|---|---|---|---|
| Age group | | | |
| < 20 years | 1 | | |
| 20-39 years | 3.405 | 0.4199–27.6184 | 0.251 |
| 40-59 years | 8.262 | 1.0424–65.4868 | 0.046 |
| ≥ 60 years | 7.000 | 0.7906–61.9762 | 0.080 |
| Male | 1.372 | 0.748–2.516 | 0.307 |
| Comorbidities | | | |
| Hypertension | 0.702 | 0.249–1.976 | 0.502 |
| Diabetes | 2.004 | 0.436–9.215 | 0.372 |
| Current smoker | 1.448 | 0.802–2.616 | 0.220 |
| Vital Signs | | | |
| Systolic blood pressure ≥ 140 mmHg | 0.571 | 0.308–1.059 | 0.075 |
| Diastolic blood pressure ≥ 90 mmHg | 0.597 | 0.316–1.129 | 0.153 |
| Heart rate > 100 /min | 0.981 | 0.511-1.881 | 0.953 |
| Respiratory rate > 16 /min | 0.247 | 0.040-1.511 | 0.151 |
| Intentional exposure | 2.246 | 1.110–4.542 | 0.024 |
| GCS score < 9 | 5.374 | 2.215–13.039 | <0.001 |
| Symptoms | | | |
| Headache | 0.439 | 0.161–1.196 | 0.108 |
| Loss of consciousness | 1.354 | 0.684–2.677 | 0.384 |
| Dyspnea | 0.426 | 0.050–3.609 | 0.434 |
| Chest pain | 2.661 | 0.367–19.306 | 0.333 |
| Laboratory findings | | | |
| White blood cell count > 10 × $10^3$/uL | 4.208 | 1.916–9.244 | <0.001 |
| BUN > 17.71 mg/dL | 5.787 | 2.922–11.501 | <0.001 |
| Creatinine > 1.2 mg/dL | 10.045 | 4.515–22.347 | <0.001 |
| Creatine Kinase > 1,000 U/L | 7.984 | 3.572–17.847 | <0.001 |
| pH < 7.2 | 5.726 | 0.508–64.487 | 0.158 |
| C-reactive protein > 9.2 mg/L | 10.503 | 4.530–24.354 | <0.001 |
| Hyperbaric oxygen therapy | 0.957 | 0.181–5.061 | 0.959 |

BMI, body mass index; BUN, blood urea nitrogen; CI, confidence interval; COHb, carboxyhemoglobin; GCS, Glasgow Coma Scale.
